# Supplementary material for: On an optimal testing strategy for workplace settings operating during the COVID-19 pandemic
Source: PLoS One. 2022 Mar 2;17(3):e0264060. doi: 10.1371/journal.pone.0264060 (PMC8890654; doi:10.1371/journal.pone.0264060)
Supplement: S2 File — (PDF) [file pone.0264060.s002.pdf]

Pseudocode. .

Each n_{ex} value to be tested

[

Each of 10,000 realisations, for j=0 to 10,000

[

Each simulation day, for i=0 to 200

[

Epidemiological Evolution

-Update infection probability, $P_I(i)$ if required.

-Draw a random number between 0-1 for all healthy individuals, if said number is $< P_I(i)$, switch the individual in question to infected status.

-Draw a second random number between 0-1 for all switched individuals above and assign each as symptomatic from the onset if said number is smaller than 0.25. If not, the infected individual is assigned as pre-symptomatic .

-Switch all infected individuals which have had this status for 15 days back to a healthy status.

-For all pre-symptomatic individuals on their 5th day of infection draw a random number between 0-1, and switch them from pre-symptomatic to symptomatic if said number is smaller than 0.6.

Intervention

-Return to active service any identified infected individuals which have completed 14 days at home.

-Apply Sanitary Checkpoint, send home for 14 days symptomatic individuals identified.

-Identify the testing sample i.e. determine if there are more than N_T attending individuals which have not been tested over the previous n_{ex} days.

-If yes, proceed to testing. If no, repeat the previous step with $n_{ex} = n_{ex} - 1$.

-Testing: test N_T individuals chosen at random from the testing sample.

-Send home for 14 days any pre-symptomatic or asymptomatic infected individuals identified.

Book keeping

-Count the number of infected individuals attending on day i i.e. IPD(i,j)=IPD(i,j)+1 for every infected individual in attendance that day.

]

Repeat for 200 days.

]

Repeat 10,000 times to gauge the intrinsic variance of the simulated situation.

-Calculate all statistics for the IPD(i) distribution over the j realisations.

]

Repeat for a range of initial n_{ex} values to be tested.
